# Prognostic Indicators for Positive Treatment Outcome After Multidisciplinary Orofacial Treatment in Patients With Somatosensory Tinnitus

**DOI:** 10.3389/fnins.2020.561038

**Published:** 2020-09-16

**Authors:** Annemarie van der Wal, Paul Van de Heyning, Annick Gilles, Laure Jacquemin, Vedat Topsakal, Vincent Van Rompaey, Marc Braem, Corine Mirjam Visscher, Steven Truijen, Sarah Michiels, Willem De Hertogh

**Affiliations:** ^1^Department of Rehabilitation Sciences and Physiotherapy, Faculty of Medicine and Health Sciences, University of Antwerp, Antwerp, Belgium; ^2^Department of Otorhinolaryngology, Antwerp University Hospital, Edegem, Belgium; ^3^Faculty of Medicine and Health Sciences, University of Antwerp, Antwerp, Belgium; ^4^Multidisciplinary Motor Centre Antwerp, University of Antwerp, Antwerp, Belgium; ^5^Department of Translational Neurosciences, Faculty of Medicine and Health Sciences, University of Antwerp, Antwerp, Belgium; ^6^Department of Human and Social Welfare, University College Ghent, Ghent, Belgium; ^7^Lab Dental Materials, University of Antwerp, Antwerp, Belgium; ^8^Special Care Dentistry, University Hospital Antwerp, Edegem, Belgium; ^9^Faculty of Medicine and Health Sciences, University of Antwerp, Antwerp, Belgium; ^10^Department of Oral Health Sciences, Academic Centre for Dentistry Amsterdam, Research Institute MOVE Amsterdam, University of Amsterdam, VU University Amsterdam, Amsterdam, Netherlands

**Keywords:** tinnitus, temporomandibular disorders, prognosis, somatic, treatment

## Abstract

**Introduction:**

Subjective tinnitus that is influenced by the somatosensory system is called somatosensory tinnitus (ST). When ST is related to the temporomandibular area, multidisciplinary orofacial treatment can reduce tinnitus severity. It is, however, unknown if we can predict this positive outcome. The aim of this study is to look for prognostic indicators that can predict a positive outcome after multidisciplinary orofacial treatment in patients with ST.

**Methods:**

Patients were included when they were diagnosed with temporomandibular-related ST and received a maximum of 18 sessions of orofacial treatment during a 9-week program. Predictors for positive treatment outcome were identified using univariate and multiple logistic regression analyses with the Tinnitus Questionnaire (TQ) and the Tinnitus Functional Index (TFI) as dependent variables.

**Results:**

The results of 101 patients were included in the analysis. Immediately after multidisciplinary orofacial treatment, a clinically relevant decrease in TQ score was significantly associated with “shorter duration of tinnitus” [odds ratio (OR) 0.99], “higher initial score on the TQ somatic subscale” (OR 1.52), and “painful palpation of the temporomandibular joint (TMJ)” (OR 2.46). After 9 weeks of follow-up, the “higher initial score on the TQ somatic subscale” remained as the sole predictor (OR 1.44). A clinically relevant decrease on TFI after 9 weeks of follow-up was predicted by “female gender” (OR 2.70), “younger age” (OR 0.96), “shorter duration of the tinnitus” (OR 0.99), “lower pressure pain thresholds (PPT) on TMJ” (OR 0.99), “lower PPT on sternocleidomastoid origin” (OR 0.99), and “better speech in noise perception” (OR 0.88). A multivariate model comprising “shorter duration of tinnitus” and “higher initial score on the somatic subscale of the TQ” correctly predicts the clinically relevant decrease in TQ score after treatment in 68.5%. A second multivariate model comprising “female gender,” “younger age,” and “shorter duration of the tinnitus” correctly predicts a clinically significant decrease on TFI after follow-up in 68.1%.

**Conclusion:**

We were able to identify various prognostic indicators. “Younger female patients” with a “shorter duration of tinnitus” and a “higher initial score on the TQ somatic subscale” appear to have the best prognosis after multimodal orofacial therapy.

## Introduction

Tinnitus or ringing in the ears is a common symptom that can have many different etiologies. It occurs in 10–15% (Baguley et al., 2013) of the adult population and is often related to hearing loss or a noise trauma. Cochlear abnormalities are considered to be the initial cause, followed by neural changes in the central auditory system that maintain the tinnitus (Baguley et al., 2013). In many patients, the perception of tinnitus is not constant and can vary (Schlee et al., 2016). This fluctuation of tinnitus can depend on various factors, such as stress (Mazurek et al., 2015), emotional state (Probst et al., 2016), anxiety (Bhatt et al., 2017), depression (Bhatt et al., 2017), cervical spine dysfunction (Michiels et al., 2015), and temporomandibular disorders (TMD) (Buergers et al., 2014). In these last two cases, tinnitus is called somatosensory (or somatic) tinnitus (ST), which is present in 12–43% of patients with subjective tinnitus (Michiels et al., 2015, 2019a). A physiological explanation for ST can be found in animal studies where connecting fibers between the dorsal cochlear nucleus (DCN) and the somatosensory nuclei are found (Kanold and Young, 2001). Through these fibers, altered cervical and temporomandibular somatosensory afference can increase the spontaneous firing rates of the DCN, causing tinnitus or altering an existing tinnitus (Levine et al., 2003; Shore et al., 2007; Shore, 2011; Wu et al., 2016). Thus, tinnitus can be evoked or modulated by inputs from the somatosensory system through increased muscle tension in the masticatory muscles or the muscles of the cervical spine or pressure on myofascial trigger points (Sanchez and Rocha, 2011; Ralli et al., 2017). This mechanism explains the larger prevalence of tinnitus in patients with temporomandibular disorders (TMD) (30.4–64%; Lam et al., 2001; Manfredini et al., 2015) compared to the general population.

As suggested by these pathophysiological models, studies investigating the effect of orofacial treatment show positive results on tinnitus severity, loudness, and annoyance (Erlandsson et al., 1991; Tullberg and Ernberg, 2006; Bosel et al., 2008; Michiels et al., 2019b; Van der Wal et al., 2020). Our recently published randomized controlled trial (RCT) showed a clinically relevant improvement after multidisciplinary orofacial treatment of tinnitus severity in 61% and of tinnitus annoyance in 46% of temporomandibular-related ST patients (Van der Wal et al., 2020). This is in accordance with previous studies (Erlandsson et al., 1991; Tullberg and Ernberg, 2006) but does not provide information on predicting factors for an individual outcome in a clinical environment. If prognostic indicators, i.e., factors that can predict treatment outcome, could be identified, the clinical success rates would dramatically improve and unnecessary treatments could be avoided.

This study, therefore, aims to identify prognostic indicators that predict a positive outcome after multidisciplinary orofacial treatment in patients with temporomandibular-related somatic tinnitus.

## Materials and Methods

### Patients

Patients were recruited from the tinnitus clinic of Antwerp University Hospital (UZA, Edegem) in Belgium. For this study, we used data collected for a RCT (80 patients) investigating the effect of multidisciplinary orofacial treatment. These data were complemented with an additional cohort of 21 patients to increase the power of our analysis (Michiels et al., 2018b; Van der Wal et al., 2020). The patients in the cohort met the same inclusion criteria and received the same treatment as the patients from the RCT. The only difference was that there was no randomization.

Before inclusion in the study, the patients got a thorough assessment by a multidisciplinary team of otolaryngologists, dentists, physical therapists, and audiologists to identify the influencing factors of their tinnitus and to exclude any objective causes (Van de Heyning et al., 2015). Only adult patients (≥18 years) were included in the study. All patients were suffering from moderate to severe chronic subjective tinnitus, attributed to the temporomandibular area, that had been stable for at least 3 months. Moderate to severe tinnitus was defined as a Tinnitus Functional Index (TFI) score between 25 and 90 (Schlee et al., 2016). Apart from tinnitus, the patients had to have a painful TMD, diagnosed according to the Diagnostic Criteria for TMD (DC-TMD) (Schiffman et al., 2014) and/or oral parafunctions.

Patients suffering from clear ontological or neurological causes of tinnitus such as Menière’s disease, progressive middle ear pathology, or intracranial pathology were excluded from the study. Patients with severe depression or anxiety disorders, as diagnosed by a psychiatrist, traumatic cervical spine or temporomandibular injury in the past 6 months, and tumors or previous surgery in the orofacial area, were likewise excluded. Patients who received orofacial treatment in the past 3 months were excluded as well.

### Intervention

The patients received a maximum of 18 sessions of orofacial physical therapy during a 9-week program. This treatment consisted of counseling regarding mouth habit reversal, bruxism, sleep hygiene, lifestyle advice, and biofeedback, massage of the masticatory muscles, and stretching exercises and relaxation therapy. In case of grinding, the orofacial physical therapy was complemented with an occlusal splint. In case the patient also suffered from cervical spine dysfunctions (as detected during clinical examination at baseline), additional cervical spine treatment (mobilizations and exercises) was added by the physiotherapist. This type of multidisciplinary orofacial treatment is currently the evidence-based treatment for the conservative management of TMD (Schiffman et al., 2014; Gil-Martinez et al., 2018).

### Outcome Measures

#### Tinnitus Assessment

The effectiveness of the treatment was measured using the Tinnitus Questionnaire (TQ) and the TFI.

The 52 questions of the TQ (Meeus et al., 2007) assess tinnitus annoyance, covering five tinnitus domains (emotional and cognitive distress, intrusiveness, auditory difficulties, sleep, and somatic complaints). The somatic subscale consists of the following three statements: (1) The noises sometimes give me a pain in the ear or head, (2) Because of the noises, I have tension in the muscles of my head and neck, and (3) The noises sometimes produce a bad headache. For each question, the level of agreement should be given on a three-point scale ranging from “true” (scoring 0) over “partly true” (scoring 1) to “not true” (scoring 2). Since two items must be counted double and 12 out of 52 items are excluded from the scoring, the total score ranges from 0 to 84, with higher scores indicating higher levels of annoyance. A decrease of 8.72 points on the TQ is considered clinically relevant (Zeman et al., 2012). The TQ showed good correlation with the Tinnitus Handicap Inventory, Tinnitus Impairment Questionnaire, and Tinnitus Functional Index (0.79–0.90) (Zeman et al., 2012; Jacquemin et al., 2019).

Apart from its use as a dependent variable, the “somatic subscale” of the TQ was used as a potential prognostic indicator.

The TFI was used to measure change in tinnitus severity (Meikle et al., 2012; Rabau et al., 2015) and consists of 25 questions covering eight tinnitus domains (intrusiveness, sense of control, cognitive interference, sleep, auditory difficulties, relaxation, quality of life, and emotional distress). Questions are answered on an 11-point Likert scale (i.e., from “no disturbance” to “maximal disturbance”), with higher scores indicating higher severity levels. A reduction of 13 points is considered clinically relevant (Meikle et al., 2012). The test – retest reliability of the TFI is good (*r* = 0.86), and the convergent validity with the Tinnitus Handicap Inventory (*r* = 0.86) and Visual Analogue Scale (VAS) (*r* = 0.75) is good (Meikle et al., 2012; Rabau et al., 2015).

### Potential Prognostic Indicators

The potential prognostic indicators we used in our analyses are clustered below into medical-history-related items, TMJ-related items, and audiological items. All included potential prognostic indicators were selected based on existing knowledge about potential influence on the outcome after treatment. More information can be found in the RCT paper and the study protocol (Michiels et al., 2018b; Van der Wal et al., 2020).

#### Medical History

At baseline, age, gender, and duration of tinnitus were inventoried apart from a specific set of ST-related questions (Table 1). These specific questions are related to the diagnostic criteria for ST (Michiels et al., 2018a), and it is generally accepted that the more criteria present, the stronger the somatic influence on tinnitus will be. Because we expect patients with a stronger somatic influence on their tinnitus to have a larger treatment effect, we included these questions as potential prognostic indicators.

**TABLE 1:** Potential prognostic indicators from medical history.

Medical history
Age in years
Gender: male/female
Duration of tinnitus in months
HADS score
NBQ score
Specific anamnestic questions:
• Modulation of tinnitus with movements of or pressure on the neck or jaw
• Modulation of tinnitus by clenching the teeth
• Modulation of tinnitus during specific postures or movements
• Tinnitus modulation with stress
• Tinnitus modulation with noise exposure
• Simultaneous increase of pain in the neck or jaw and tinnitus
• Temporal coincidence of the onset of pain complaints in the jaw and tinnitus
• Grinding of the teeth during the day or night
• Clenching of the teeth during the day or night
• Temporal headache related to temporomandibular disorders

**HADS, Hospital Anxiety and Depression Scale; NBQ, Neck Bournemouth Questionnaire.**

Next, the scores of three questionnaires were added as potential prognostic indicators.

The Hospital Anxiety and Depression Scale (HADS) was added to identify the presence of anxiety and depression (Zigmond and Snaith, 1983; Snaith and Zigmond, 1986). The presence of anxiety or depression was selected because these conditions strongly affect tinnitus severity and annoyance and can negatively influence the outcome after treatment.

The HADS is specifically developed for patients in non-psychiatric hospital clinics and contains two subscales, an Anxiety subscale (HADS-A) and a Depression subscale (HADS-D). Each subscale contains seven questions that are answered on a four-point (0–3) scale. The scores range from 0 to 21 (Zigmond and Snaith, 1983; Snaith and Zigmond, 1986; Snaith, 2003). A score greater than or equal to 11 indicates the potential presence of an anxiety or depression disorder. The HADS has been found to be reliable as a first indication of a depression or anxiety disorder in somatic, psychiatric, and primary care (Bjelland et al., 2002).

Additionally, the presence of hyperacusis, objectified using the Hyperacusis Questionnaire (HQ) (Khalfa et al., 2002), was included, as the presence of hyperacusis, in addition to tinnitus, might negatively influence the outcome after treatment. Hyperacusis and tinnitus complaints are highly intertwined, and patients whose tinnitus complaints improve might not always notice this improvement when their hyperacusis remains unchanged (Kusdra et al., 2018).

The HQ consists of 14 questions answered on a four-point scale from “No” (scoring zero point), “Yes, a little” (scoring one point), and “Yes, quite a lot” (scoring two points) to “Yes, a lot” (scoring three points). Total scores on the HQ range from 0 to 42, and a score from 28 points upwards indicates the presence of hyperacusis.

The presence of neck complaints, identified as a score of 13 points or more on the Neck Bournemouth Questionnaire (NBQ), was added because these neck complaints can influence temporomandibular complaints or tinnitus directly.

The NBQ is used to assess self-reported pain intensity, limitations in activities of daily living, depression, and self-control. The test–retest reliability of the NBQ is moderate (intraclass correlation coefficient, ICC = 0.65), and the construct validity with the Neck Disability Index is acceptable (*r* = 0.50) (Bolton and Humphreys, 2002).

#### Temporomandibular Assessment

Apart from the potential prognostic indicators retrieved from medical history, a set of baseline TMD tests was added to investigate the importance of the degree or nature of TMD in the prediction of positive treatment outcome. An overview of the assessment is presented in Table 2.

**TABLE 2:** Potential prognostic indicators from the temporomandibular assessment.

Temporomandibular assessment
TMD pain screener questionnaire (Gonzalez et al., 2011)
Orofacial assessment:
• Measurement of active mouth opening (including questioning of any pain sensation) according to the DC/TMD (Schiffman et al., 2014)
• Tenderness on palpation of the jaw muscles and temporomandibular joint according to the DC/TMD (Schiffman et al., 2014)
• Pain provocation testing during static and dynamic movements as described by Visscher et al. (2007)
• Diagnosis of arthralgia or myalgia according to the DC/TMD (Schiffman et al., 2014)
Mean pressure pain threshold as described by Visscher et al. (2004):
• On the anterior portion of the temporalis muscle
• On the muscle belly of the masseter
• On the insertion of the sternocleidomastoid muscle (just below the mastoid process)
• On the lateral pole of the temporomandibular condyl
• On the muscle belly of the tibialis anterior muscle (7 cm below the tibial tuberosity)

**TMD, Temporomandibular Disorders; DC/TMD, Diagnostic Criteria for Temporomandibular Disorders*.*

Firstly, the TMD pain screener was completed as an indication for the presence of painful TMD. The TMD pain screener consists of six questions, and the total scores range between 0 and 7. Scores of three points or more are suspected to have a painful TMD based on the Diagnostic Criteria/TMD (DC/TMD) (Schiffman et al., 2014). The TMD pain screener has excellent sensitivity and specificity (0.99 and 0.95–0.98) to detect painful TMD, and the reliability is good (ICC, 0.79).

Secondly, an orofacial assessment was performed according to the standardized protocol of the DC/TMD (Schiffman et al., 2014). This assessment comprises the measurement of active mouth opening (including questioning of any pain sensation), tenderness on palpation of the jaw muscles, and temporomandibular joint and pain provocation during static and dynamic movements. Based on the DC/TMD protocol, the assessor was able to diagnose the presence of TMD and give an indication if this TMD was mostly articular, mainly muscular, or combined articular and muscular in origin.

Lastly, pressure pain thresholds (PPTs) were measured using a handheld algometer (Somedic AB, farsta, Sweden). These measurements were added because they give us an indication on the presence of central sensitization of the patient’s TMD pain complaints (Jespersen et al., 2013; Coronado et al., 2014). Patients showing clear central sensitization are known to react less to the orofacial treatment that we used is this study, which might negatively influence the prognosis.

The PPTs were measured on the following locations: the anterior portion of the temporalis muscle, the muscle belly of the masseter, the insertion of the sternocleidomastoid muscle (on the mastoid process), the lateral pole of the temporomandibular condyl, and the muscle belly of the tibialis anterior muscle. The patients were instructed to indicate the exact moment the sensation changed from pressure to pain. The test was repeated three times with a 1 min rest between the tests, and average values were calculated for each area (Visscher et al., 2004).

#### Audiological Assessment

The potential prognostic indicators from the audiological assessment are presented in Table 3.

**TABLE 3:** Potential prognostic indicators from the audiological assessment.

Audiological assessment
Pure tone audiometry: Fletcher index low according to the current clinical standards (ISO 8253-1, 1989)
Speech reception threshold in noise, using the Leuven Intelligibility Sentence Test (van Wieringen and Wouters, 2008)
Psychoacoustic tinnitus analyses described in our study protocol (Michiels et al., 2018b):
• Type of tinnitus (e.g., pure tone, noise)
• Tinnitus pitch
• Tinnitus loudness (expressed in dB SL)

As a first item, hearing loss was added because hearing loss is, in many patients, the major cause for their tinnitus. The presence of (severe) hearing loss might therefore negatively influence the outcome after treatment. Hearing loss was objectified using pure tone audiometry. Air conduction thresholds were measured at 125, 250, 500 Hz, 1, 2, 3, 4, 6, and 8 kHz according to current clinical standards (ISO 8253-1, 1989). Based on these results, the Fletcher Index low was calculated as the mean of pure tones at 500 Hz, 1, and 2 kHz.

Secondly, speech in noise tests was added because, even in patients without hearing loss, speech perception in noise is often decreased in patients with tinnitus. The presence of these speech perception problems might negatively influence the outcome after treatment, especially if this treatment is not directed to influence the patients’ hearing.

The Leuven Intelligibility Sentence Test (LIST) (van Wieringen and Wouters, 2008), a Dutch sentence test, was used. The LIST consists of 35 lists of 10 sentences that are a reflection of daily communication and are of equivalent difficulty. An adaptive procedure is used with noise at a fixed level of 65 dB SPL. The procedure starts at a signal-to-noise ratio (SNR) of 0 dB, which means that speech and noise are presented equally loud (65 dB SPL). Subsequently, the intensity level within a list of sentences is varied in steps of 2 dB adaptively in a one-down (when the keywords in the sentence are correctly repeated), one-up (when the keywords in the sentence are incorrectly repeated) procedure to determine the 50% correct identification point which is called the speech reception threshold, expressed in dB SNR. Before starting the actual procedure, one list will be performed as a training list.

Finally, we added psychoacoustic tinnitus properties because previous research showed that patients with low-pitched somatic tinnitus were more likely to benefit from cervical spine treatment (Michiels et al., 2017). The audiologist measured the type of tinnitus, tinnitus pitch, and tinnitus loudness. For identifying the type of tinnitus, the patient was asked whether he/she perceived a pulsatile or non-pulsatile tinnitus, whether the tinnitus was present constantly or intermittent, and whether the tinnitus sound is a pure tone, a noise, or polyphonic (a mixture of different sounds). The tinnitus pitch measurement is a technique where the audiologist identifies the pitch of the tinnitus by presenting a set of pure tones or noises (depending on the type of tinnitus) to the patient. This procedure is repeated until the exact match is obtained. Finally, tinnitus loudness matching is performed.

### Statistics

The relationship between the presence of a clinically relevant reduction on TQ and TFI after orofacial treatment and potential prognostic indicators was evaluated using binary logistic regression analyses.

Before the actual analysis, the normality of the data was assessed using the Kolmogorov–Smirnov test. Next, correlations between potential prognostic indicators and the clinical outcome on TQ and TFI were calculated using Pearson or Spearman correlation coefficients, depending on the normality of the data. Potential prognostic indicators that correlated significantly (*p* < 0.10) with the clinical outcome were included in the logistic regression analysis. Significance levels were chosen to allow a broad screening for potential prognostic indicators, as suggested by Hicks et al. (2005). Correlation coefficients were additionally used to avoid multicollinearity and shared variance (*r* > 0.80) between the different potential prognostic indicators.

Then, a univariate logistic regression analysis was performed. As dependent variable, the dichotomous variables of clinically relevant reduction on TQ and TFI were used (obtained yes/no). These variables were computed based on a decrease of 8.72 points for the TQ and 13 points for the TFI (Meikle et al., 2012; Zeman et al., 2012). In total, four different variables were used as dependent variables: clinically relevant reduction described as a decrease of 8.72 on the TQ scale immediately after treatment and after 9 weeks of follow-up and a clinically relevant reduction described as a decrease of 13 points on the TFI immediately after treatment and after 9 weeks of follow-up. As potential prognostic indicators, the characteristics described in Tables 1–3 that correlated significantly with the dependent variable (*p* < 0.10) were used. Odds ratios, 95% confidence intervals, and *p*-values were calculated for every potential prognostic indicator.

Afterward, a multivariate model for the prediction of a clinically relevant improvement on TQ and TFI was created using multivariate logistic regression analyses. For these analyses, only the strongest prognostic indicators (*p* < 0.10) from the univariate analyses were retained. In case of multicollinearity or shared variance between two or more indicators, only the strongest indicator was entered in the multivariate logistic regression analysis.

## Results

In total, data from 101 patients were included in the analysis. All patients suffered from moderate to severe tinnitus, with an average TQ score of 38 points (*SD* 16) and an average TFI score of 53 points (*SD* 17). Most patients (81.2%) were diagnosed with myalgia, and 24.8% of the patients had both myalgia and arthralgia according to the DC-TMD (Schiffman et al., 2014). In total, 35.6% of the patients had hearing loss, using the pure tone audiometry as main hearing test to determine if the patient’s hearing levels fall within normal limits according to age. In addition to the physical therapy treatment, 54% of the patients received an occlusal splint (48% female/52% male). An overview of the patient characteristics is summarized in Table 4.

**TABLE 4:** Patients’ characteristics at baseline (*n* = 101).

Characteristics	Mean and standard deviation
Age (*SD*)	47 years (14)
Gender: % female/male	49/51%
Mean duration of tinnitus	64 months (89)
% subacute tinnitus (3–6 months)	25.7%
% chronic tinnitus (>6 months)	74.3%
TFI score (*SD*)	53 (17)
TQ score (*SD*)	38 (16)
VAS mean loudness, left ear (*SD*)	48 (29)
VAS mean loudness, right ear (*SD*)	51 (29)
Hyperacusis Questionnaire	18 (8)
% HQ score ≥ 8	12.9%
% HQ score <28	87.1%
HADS (anxiety) (*SD*)	9 (4)
HADS (depression) (*SD*)	6 (5)
% diagnosed with TMD myalgia	81.2%
% diagnosed with TMD arthralgia	23.8%
% diagnosed with both myalgia and arthralgia	24.8%
% with bruxism	91%
TMD pain screener (% < 3/% ≥ 3)	41%/59%
NBQ (*SD*)	23 (14)
% with hearing loss	35.6%
Fletcher index low, left	12 (15)
Fletcher index low, right	11 (12)
Tinnitus loudness dBHL, left	32 dB (21)
Tinnitus loudness dBHL, right	29 dB (20)
Tinnitus loudness dBSL, left	8 dB (9)
Tinnitus loudness dBSL, right	9 dB (13)
Mean SPIN (signal to noise ratio)	−3 (5)

**SD, standard deviation; TFI, Tinnitus Functional Index; TQ, Tinnitus Questionnaire; VAS, Visual Analogue Scale; HADS, Hospital Anxiety and Depression Scale; TMD, temporomandibular disorders; NBQ, Neck Bournemouth Questionnaire; dBHL, decibels hearing level; dBSL, decibels sensation level; SPIN, speech in noise.**

### Prognostic Indicators

Table 5 shows the statistically significant prognostic indicators for a clinically relevant reduction in TQ score after treatment and after 9 weeks of follow-up.

**TABLE 5:** The statistically significant prognostic indicators of clinically relevant improvement on the Tinnitus Questionnaire.

Variable	Tinnitus questionnaire					
	
	Univariate regression analysis					
	
	After treatment			After 9-week follow-up		
		
	OR	95%	*P*	OR	95%	*P*
Duration of tinnitus	0.99	0.98–0.99	0.03			
Somatic subscale of the Tinnitus Questionnaire	1.52	1.16–1.99	0.002	1.44	1.12–1.84	0.004
Palpation of the temporomandibular joint	2.46	1.00–6.04	0.05			

Table 6 shows the statistically significant prognostic indicators for a clinically relevant reduction in TFI score after 9 weeks of follow-up. No statistically significant associations were found for clinically relevant improvement immediately after treatment.

**TABLE 6:** Prognostic indicators of clinically relevant improvement on the Tinnitus Functional Index.

Variable	Tinnitus functional index		
	
	Univariate logistic regression analysis		
	
	After 9 week follow-up		
	
	OR	95%	*P*
Female gender	2.70	1.12–6.21	0.02
Age	0.96	0.94–0.99	0.02
Duration of the tinnitus	0.99	0.98–0.99	0.008
Mean PPT TMJ	0.99	0.99–1.00	0.03
Mean PPT SCM	0.99	0.99–1.00	0.04
Mean SPIN	0.88	0.77–0.99	0.04

**TMJ, Temporomandibular Joint; PPT, Pain Pressure Threshold; SCM, Sternocleidomastoid muscle; SPIN, speech in noise*.*

The multivariate logistic regression analysis, based on the clinically relevant change in TQ score after treatment, created a model comprising two characteristics: “duration of tinnitus” and “a higher initial score on the TQ somatic subscale.” This model correctly predicts the outcome on TQ in 68.5% (Table 7).

**TABLE 7:** Multiple regression analyses based on the clinically relevant change in Tinnitus Questionnaire (TQ) score after multidisciplinary orofacial treatment.

	Multiple regression analyses on TQ		
	
	score after treatment		
	
	OR	95% Cl	*P*
Duration of tinnitus	0.99	0.98–0.99	0.03
Somatic subscale of the Tinnitus Questionnaire	1.57	1.19–2.08	0.002

Additionally, the multivariate binary logistic regression analysis, based on the clinically relevant change in TFI score after follow-up, created a model consisting of three items: “age,” “female gender,” and “duration of tinnitus.” This model correctly predicts the outcome on TFI in 68.1% (Table 8).

**TABLE 8:** Multiple regression analyses for the Tinnitus Functional Index (TFI) score at 18 weeks.

	Multiple regression analyses		
	
	on TFI score after follow-up		
	
	OR	95% Cl	*P*
Age	0.96	0.93–0.99	0.01
Female gender	3.24	1.27–8.26	0.04
Duration of tinnitus	0.99	0.98–0.99	0.03

## Discussion

The aim of this study was to identify prognostic indicators that can predict a positive outcome after multidisciplinary orofacial treatment in patients with temporomandibular-related somatic tinnitus.

We were able to identify three prognostic indicators for a positive treatment effect immediately after treatment and seven for a positive treatment effect after 9 weeks of follow-up. A prognostic model, with two variables, that allowed us to correctly predict a positive outcome on TQ in 68.5% of patients was made. Additionally, a second prognostic model, with three variables, that correctly predicts a positive outcome on TFI in 68.1% of patients was created.

The most important predictors from medical history were “female gender” [odds ratio (OR) 2.70] and “a higher score on the somatic subscale of the TQ” (OR 1.52). From the temporomandibular assessment, “painful palpation of the TMJ” (OR 2.46) was retrieved as a prognostic indicator. Furthermore, a “better score on the speech in noise test” (OR 0.88) was found to be an important prognostic indicator from audiological assessment. The clinical relevance of other significantly associated prognostic indicators is limited because their ORs are very close to 1.

The fact that patients with a higher initial score on the somatic subscale of the TQ perform better after orofacial treatment seems logical for two reasons. First, a higher somatic subscore on TQ might be an indication that patients have more TMD complaints at baseline and greater alterations in somatosensory afference that can influence tinnitus. Since our orofacial treatment aims to decrease TMD complaints and normalize somatosensory afference, a larger improvement can be expected in patients with more complaints at baseline. Second, a higher initial score on one subscale of the TQ gives more room for a decrease in the total score after treatment, especially since our treatment aims to decrease the somatic burden. This explanation is in accordance with the most important prognostic indicator from the temporomandibular assessment. A painful palpation of the TMJ might suggest a higher somatic burden that can explain a better outcome after multidisciplinary orofacial treatment.

Shorter duration of tinnitus is a positive prognostic indicator for clinically relevant improvement on both TQ and TFI. This is in line with the findings of Ariizumi et al. (2010), who also found that subjective tinnitus patients with a shorter duration of tinnitus performed better after tinnitus retraining therapy with a sound generator. It must be noted though that “shorter duration of tinnitus” only predicts a better short-term outcome on TQ.

When looking at improvement on TFI, young females are more likely to benefit from orofacial treatment. This observation might be explained by the fact that TMD is more prevalent in females, also in female tinnitus patients (Manfredini et al., 2006; Vielsmeier et al., 2011; Bagis et al., 2012; Guarda-Nardini et al., 2012; Ward et al., 2015; Ralli et al., 2018), and our treatment might be more effective in patients with painful TMD than in patients with oral parafunctions without pain complaints.

Additionally, patients with better SPIN scores were more likely to benefit from orofacial treatment. A good performance on the SPIN test indicates that there is no significant hearing loss. In these patients, other factors, such as TMJ dysfunctions, may have a larger influence on tinnitus. This can explain their better performance in our study.

On the other hand, several potential prognostic indicators that were assumed to have a large influence, based on previous studies, were not able to predict the outcome after orofacial treatment in our study. Surprisingly, a patient’s ability to modulate tinnitus was not identified as a prognostic indicator. Although the ability to modulate tinnitus is an important diagnostic criterion when combined with other criteria, the Delphi panel in 2018 (Michiels et al., 2018a) already warned that tinnitus modulation is not specific for somatic tinnitus. The results of our study confirm this statement, since the ability to modulate the tinnitus did not predict a positive treatment outcome.

A second item that was thought to be an important prognostic indicator is the presence of anxiety or depression symptoms. Unexpectedly, lower scores on the HADS did not predict a better outcome after treatment. This might be caused by consistent relatively high HADS scores in all our patients. These relatively high scores can be expected in our population because anxiety and depression are associated not only with tinnitus but also with the occurrence of temporomandibular disorders (Bonjardim et al., 2005; Mongini, 2007; Mongini et al., 2007).

Additionally, our results indicate that patients showing more severe local pain complaints in the temporomandibular area at baseline are more likely to benefit from orofacial treatment. This predictive value of local orofacial pain perception was not associated with a difference in central pain sensitization because PPTs on the anterior tibialis muscle did not significantly differ between the clinically improved and the not clinically improved patients. This is in contrast with the current literature, which indicates that patients with central sensitization are less likely to benefit from applied orofacial treatment (Dahan et al., 2015; Harper et al., 2016; Monaco et al., 2017; La Touche et al., 2018).

Finally, it could be expected that the absence of hyperacusis would be a prognostic indicator for positive treatment outcome. Patients with higher scores on the hyperacusis questionnaire present stronger auditory hypersensitivity and might be more difficult to treat (Hiller and Goebel, 2006). It should be specified that we only calculated the continuous data of the hyperacusis questionnaire because there is no consensus among authors as to what should be an appropriate cutoff for classifying hyperacusis at the moment (Fackrell et al., 2015; Oishi et al., 2017).

It must be noted that some of the identified prognostic indicators might secondarily be influenced by the fact that the treatment in our study was tailored to each patient’s individual needs. Some patients received additional cervical spine treatment and 54% of the patients received an occlusal splint. Consistent differences in the use of splints between females and males, for instance, would be a potential explanation of the better outcome in females. After a thorough *post hoc* investigation though, no such consistent differences could be identified.

Based on the current analysis, we cannot be sure to what extent the changes in TMD are directly responsible for the decrease in tinnitus severity and annoyance. Other factors that are part of the orofacial treatment (for example, counseling for stress reduction) may also have a direct influence on the tinnitus complaints. Future research should investigate if there is a mediating effect of reduction in TMD pain on the improvement of tinnitus severity and annoyance.

## Conclusion

We were able to identify various prognostic indicators. “Younger female patients” with a “shorter duration of tinnitus” and a “higher initial score on the TQ somatic subscale” are the most consistent indicators with the highest predictive value. The presented prognostic indicators can be used to increase the clinical success rates of orofacial treatment on tinnitus severity and annoyance by better targeted referral. However, these results need to be confirmed in RCTs using these prognostic indicators as inclusion criteria.

## Data Availability Statement

The raw data supporting the conclusions of this article will be made available by the authors, without undue reservation.

## Ethics Statement

The studies involving human participants were reviewed and approved by the Ethics Committee of the Antwerp University Hospital (reference number B300201730825). The patients/participants provided their written informed consent to participate in this study.

## Author Contributions

AW performed the analysis, collected the data, and wrote the manuscript. PV conceived and designed the study protocol, contributed to writing the manuscript, and took charge of patient inclusion. AG collected the data, contributed to writing the manuscript, and took charge of patient inclusion. LJ collected the data and contributed to writing the manuscript. VT and VV contributed to writing the manuscript and took charge of patient inclusion. MB contributed to writing the manuscript. CV conceived and designed the study protocol and contributed to writing the manuscript. ST performed the analyses and contributed to writing the manuscript. SM performed the analysis, collected the data, conceived and designed the study protocol, and wrote the manuscript. WD performed the analysis, conceived and designed the study protocol, and contributed to writing the manuscript. All authors contributed to the article and approved the submitted version.

## Conflict of Interest

The authors declare that the research was conducted in the absence of any commercial or financial relationships that could be construed as a potential conflict of interest.
